# Decreased Expression of TMEM173 Predicts Poor Prognosis in Patients with Hepatocellular Carcinoma

**DOI:** 10.1371/journal.pone.0165681

**Published:** 2016-11-04

**Authors:** Yang Bu, Fang Liu, Qing-An Jia, Song-Ning Yu

**Affiliations:** 1 Hepatobiliary Surgery, General Hospital of Ningxia Medical University, Yinchuan, China; 2 Medical Examination Center, General Hospital of Ningxia Medical University, Yinchuan, China; 3 Hepatobiliary Surgery, The First Affiliated Hospital of Xi’an JiaoTong University, Xi’an, China; University of North Carolina at Chapel Hill School of Medicine, UNITED STATES

## Abstract

Hepatocellular carcinoma (HCC) is one of the most lethal cancer types, and chronic infection with Hepatitis B Virus (HBV) is identified as the strongest risk factor for HCC. Transmembrane Protein 173 (TMEM173) is a pattern recognition receptor which functions as a major regulator of the innate immune response to viral and bacterial infections. However, the prognostic value of TMEM173 in HCC remains elusive. Thus, we aimed to evaluate the potential prognostic significance of TMEM173 expression in HCC patients following curative resection. Immunohistochemistry was used to detect TMEM173 expression in 96 HCC patients. We found that TMEM173 protein expression was remarkably decreased in tumor tissues compared to non-tumor tissues, and that TMEM173 staining intensity was inversely correlated with tumor size, tumor invasion TNM stage and overall survival (OS) in HCC patients. Multivariate analysis supported TMEM173 as an independent prognostic factor, and identified that combining TMEM173 expression with TNM stage showed better prognostic efficiency for OS in HCC patients. In summary, TMEM173 was discovered having an independent prognostic value and may serve as a potential immunotherapeutic target for HCC.

## Introduction

Hepatocellular carcinoma (HCC) is one of the most common primary live cancers causing significant cancer-related mortalities every year [1]. Although treatments such as surgery and chemotherapy have currently improved, most patients still face a dismal prognosis[2]. Traditional tumor-node-metastasis (TNM) classification systems provide a basic prognostic model but still have limited capacity to differentiate outcomes when considering the asymptomatic nature and limited detection of early stage HCC[3]. Therefore, it is still of particular urgency to establish a better prognostic model and find prognostic biomarkers with higher sensitivity, specificity and accuracy.

Transmembrane Protein 173 (TMEM173), also named stimulator of interferon genes (STING), is a transmembrane protein localized in endoplasmic reticulum[4]. Previous studies revealed that it played an important role in the innate immune response to viral and bacterial infections via regulating type-I IFN signaling and thereby innate immunity[5]. It was identified as one of the critical adaptor proteins for cytosolic DNA sensing pathways [6]. TMEM173 has been reported to bind, through its globular carboxyl-terminal domain, cyclic dinucleotides such as cyclic di-AMP, cyclic di-GMP or cyclic di-GMP-AMP produced by virus or bacteria and thus facilitate downstream interaction with cytosolic kinase TBK1 and activation of interferon regulatory factor 3 (IRF3) or signal transducer and activator of transcription factor 6 (STAT6) [5–9]. Then activated IRF3 or STAT6 translocated into nuclei to induce interferons among other cytokines [9]. These data proved that TMEM173 functioned as a pattern recognition receptor in detection of cytosolic nucleic acids and mediation of related signal transduction.

Recently, the suppressive role of TMEM173 in tumorigenesis was suggested in several cancer types, such as prostate cancer, colorectal carcinoma and melanomas [10–12]. However, its function in HCC remains largely unknown. Since one of the strongest risk factors for HCC is chronic Hepatitis B Virus (HBV) infection [13], TMEM173 might be involved in viral detection and subsequently contributes to tumorigenesis and progression of HCC. Therefore, to study the role of TMEM173 in HCC, we evaluated the expression of TMEM173 in 96 HCC samples and tried to elucidate its correlation with tumor development and prognosis. In addition, we explored whether combination of TNM stage and TMEM173 expression could establish a better prognostic model for HCC patients.

## Materials and Methods

### Patients and specimens

For tissue microarray detection, tumor specimens including 96 HCC tissues and 96 adjacent non-tumor tissues were obtained from patients who underwent surgical resection without preoperative treatment from 2004 to 2008, at Department of General Surgery, General Hospital of Ningxia Medical University (Ningxia, China). The clinicopathological and baseline demographic characteristics of the patients, including age, gender, tumor size, tumor site and tumor stage were retrospectively collected. Tumor stages were histologically classified according to the 7th Edition of the American Joint Committee on Cancer TNM classification. OS was calculated from the date of surgery to the date of death (or the last follow-up). Follow-up was terminated in December 2011. Ethical approval was obtained from the Research Ethics Committee of The General Hospital of Ningxia Medical University and written consent was obtained from each patient.

### GEO datasets

These data are publically available from GEO database (accession number: GSE54236, GSE36411). The relative mRNA expression was achieved through the Oncomine database (https://www.oncomine.org/resource/login.html).

### Tissue microarray and immunohistochemistry

Tissue microarrays and immunohistochemistry analysis were performed as previously described, and the tissue microarray was established with formalin-fixed paraffin-embedded surgical specimens. Primary anti-TMEM173 antibody (Abcam, Cambridge, MA) was used for immunohistochemistry staining. The intensity of immunostaining was evaluated by two independent pathologists without the knowledge of clinicopathological data. The staining intensity was sorted by 0 (negative), 1 (weak), 2 (moderate) and 3 (strong). Depending on the staining extent, the area was categorized as 0 (< 5%), 1 (5%-25%), 2 (26%-50%), 3 (51%-75%) and 4 (>75%). The staining score was computed by multiply staining intensity with staining area, yielding a series of results ranged from 0 to 12.

### Statistical analysis

ROC analysis was conducted to select the optimum cut-off value of the staining score to dichotomize the patients into low and high groups. Comparisons between TMEM173 expression and clinicopathologic variables were evaluated using chi-square test. Survival curves were conducted by Kaplan-Meier method and compared by log-rank test. The Cox proportional hazards regression model was applied to evaluate multivariate analyses, and those statistically significant characteristics in univariate analysis were used to perform multivariate analysis. Nomogram was set to construct the prognostic model. Calibration plot was used to evaluate the prognostic accuracy of the models. ROC analysis was conducted to compare the sensitivity and specificity for the prediction of OS by the prognostic models. Differences between two groups were tested with Student’s two-tailed t test. All statistical tests were two-tailed and differences were considered significant at level of < 0.05. Data were analyzed using IBM SPSS Statistics 22.0 (SPSS, Chicago, IL, USA) and R software 3.2.2 with the “rms” package (R Foundation for Statistical Computing, Vienna, Austria).

## Results

### Down-regulated TMEM173 was detected in HCC

**Fig 1:**
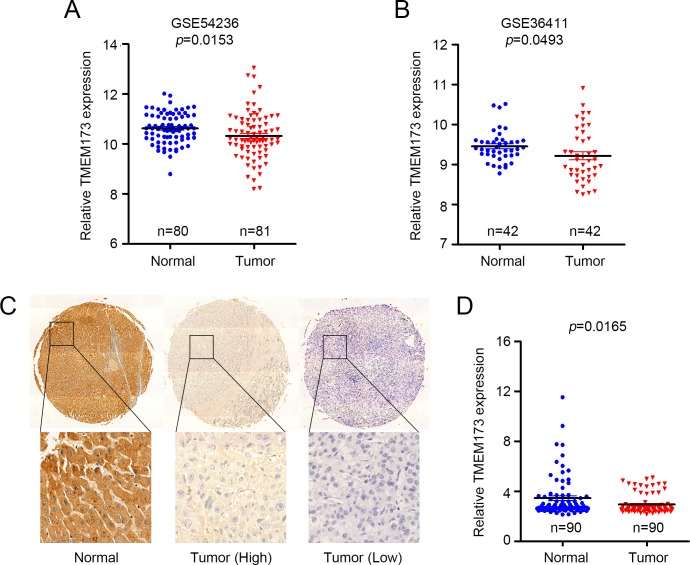
The expression patterns of TMEM173 in HCC tissues. (A-B) Relative expression of TMEM173 mRNA in HCC and normal liver tissues in GSE54236 (A) and GSE36411 (B). (C) Representative IHC staining images of TMEM173 and its regional magnification in HCC tissues and non-tumor tissues. Scale bar = 200 μm. (D) IHC score of TMEM173 expression in HCC tissues and non-tumor tissues.

To understand whether TMEM173 was involved in HCC carcinogenesis, we first examined the mRNA expression of TMEM173 in HCC tissues from reported GEO datasets. We found that the TMEM173 mRNA expression was decreased in tumor tissues in both GSE54236 [14] (P = 0.045) and GSE26411 [15] (P<0.001) datasets (Fig 1A and 1B). We next investigated the protein expression of TMEM173 in HCC samples and adjacent non-tumor tissues. Immunohistochemically (IHC) assay revealed that the protein expression of TMEM173 was down-regulated in HCC samples compared to peripheral non-tumor tissues (P<0.001) (Fig 1C and 1D).

### Correlation between TMEM173 expression and clinicopathological features in HCC patients

To further evaluate the protein level of TMEM173 and clinicopathological features, we analyzed the correlation between TMEM173 expression and clinicopathological features in 96 HCC samples. According to the results conducted by receiver operating characteristic (ROC) curve analysis, IHC score of 6 was determined as the cut-off to dichotomize the patients into TMEM173 low group (score, 0–6; n = 24) and TMEM173 high group (score, 7–12; n = 72). The correlation between TMEM173 expression and clinicopathological variables in HCC patients was analyzed with the chi-square test, and the result was listed in Table 1. Among the variables, low expression of TMEM173 in HCC was correlated with tumor venous infiltration (P = 0.041) and advanced TNM stage (P = 0.004) (Table 1). Other clinicopathologic variables showed no significant correlation with TMEM173 expression.

**Table 1:** Relationship between TMEM173 expression and clinicopathological characteristics in patients with HCC.

		TMEM173 expression		
		High	Low	
Variables	No.	No. (%)	No. (%)	*P*-value
**Age (year)**				0.820422
< 60	48	14(29%)	34(71%)	
≥ 60	48	13(27%)	35(73%)	
**Gender**				0.37796
Male	57	21(37%)	36(63%)	
Female	39	11(28%)	28(72%)	
**Tumor size (cm)**				0.124144
≤5	52	18(35%)	34(65%)	
>5	44	9(20%)	35(80%)	
**Child-Pugh’s Grade**				0.079568
A	80	31(39%)	49(61%)	
B	16	10(63%)	6(38%)	
**Venous invasion**				0.041134*
Absent	81	31(38%)	50(62%)	
Present	15	10(67%)	5(33%)	
**Distant metastasis**				0.052283
Absent	91	5(5%)	86(95%)	
Present	5	1(33%)	2(67%)	
**TNM stage**				0.004324*
I	45	20(44%)	25(56%)	
II-IV	51	9(18%)	42(82%)	

Abbreviations: TNM = tumor node metastasis. n.s. is not significant; ^*^P < 0.05 is significant.

### Correlation between TMEM173 expression and overall survival in HCC patients

**Fig 2:**
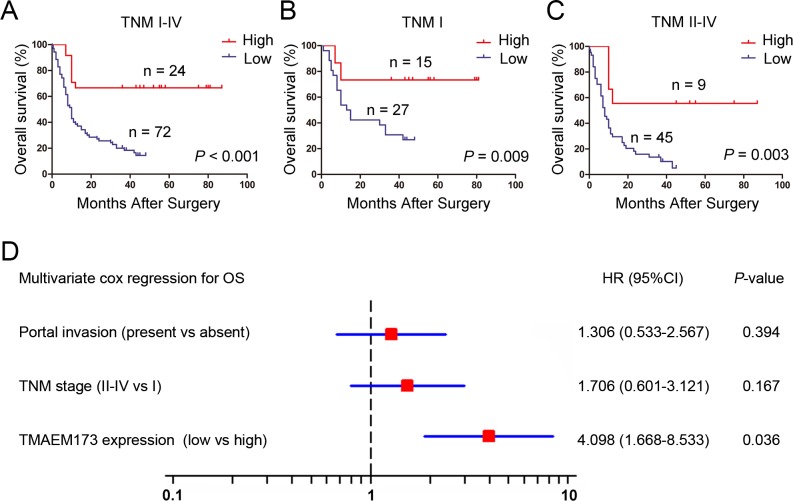
The predictive value of TMEM173 expression in patients with HCC. (A-C) Kaplan-Meier survival analysis showing the relationship between TMEM173 expression and overall survival in all patients (A), patients at TNM I stage (B) and patients at TNM II-IV stage (C). (D) Cox multivariate analysis identified the independent prognostic factors for overall survival for patients with HCC.

We next explored the relationship between TMEM173 expression and overall survival using Kaplan-Meier analysis. The results demonstrated that patients with TMEM173 low expression showed poorer overall survival than those with TMEM173 high expression (*P* < 0.001) (Fig 2A). To further evaluate the efficiency of TMEM173 expression in patients with different TNM stages, we divided the patients into early (I) and advanced (II -IV) groups. In both the TNM I and TNM II +IV subgroups, low expression of TMEM173 showed statistically significant value in predicting the outcomes of HCC patients (Fig 2B and 2C). These data suggest that TMEM173 expression is inversely correlated with overall survival for patients with HCC.

### TMEM173 expression is identified as an independent prognosticator in patients with HCC

We also conducted univariate Cox analysis to identify the prognostic significance of clinicopathological factors for overall survival. Tumor venous invasion (P = 0.001), Cirrhosis (P = 0.039), TNM stage (P = 0.047), and TMEM173 expression (P = 0.008) were found to be risk factors for survival in HCC patients (Table 2). Further adjustment of covariate factors using multivariate Cox analysis identified down-regulated TMEM173 (P < 0.001) as an independent risk factor for HCC (Fig 2D). These data indicate that low expression of TMEM173 has an independent prediction value in prognosis of HCC patients.

**Table 2:** Univariate and multivariate Cox regression analysis of clinicopathological characteristics influencing the overall survival of HCC patients.

Variables	Univariate Analysis		Multivariate Analysis	
	HR (95% CI)	P-value	HR (95% CI)	P-value
**Age**				
≤60 years vs. >60 years	0.68 (0.30–1.37)	0.085		n.s.
**Gender**				
Male vs. Female	1.44 (0.49–5.36)	0.144		n.s.
**Cirrhosis**				
Yes vs. No	1.97 (0.89–5.64)	0.039*		n.s.
**TNM Stage**				
I vs. II & III	0.34 (0.12–0.88)	0.047*		n.s.
**Venous Invasion**				
VI vs. NI	0.52 (0.21–0.89)	0.001*		n.s.
**Child-Pugh’s Grade**				
A vs. B	2.67 (1.25–5.03)	0.077		n.s.
**Tumor size**				
≤5 cm vs. >5 cm	2.56 (0.69–5.21)	0.055		n.s.
**TMEM173 expression**				
Low vs. High	3.87 (1.23–8.99)	0.008*	3.11 (1.57–7.66)	0.036*

Abbreviation: 95% CI = 95% confidence interval; HR = hazard ratio; TNM = tumor node metastasis. n.s. is not significant

* *P* < 0.05 is significant.

### Combination of TMEM173 expression and TNM stage generates a better prognostic model for overall survival of HCC patients

**Fig 3:**
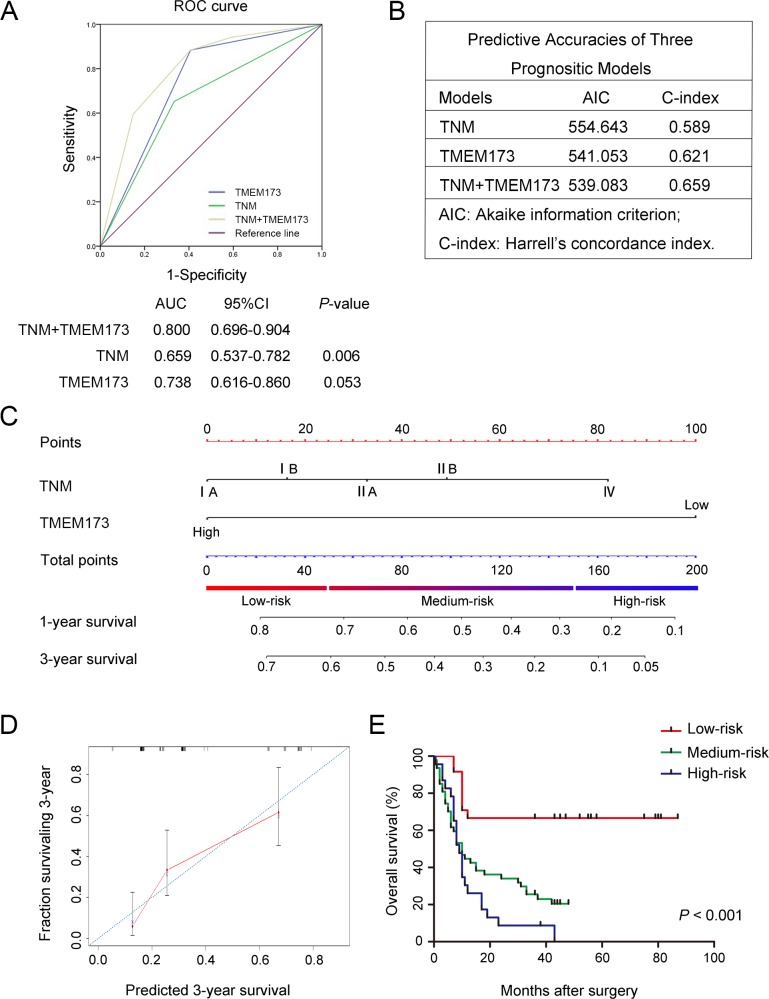
Combination of TMEM173 expression with TNM stage generates a better predictive model for overall survival of HCC patients. (A) ROC curve analysis of the sensitivity and specificity of the predictive value of the TNM stage model, TMEM173 model and the combined model. (B) AIC and Harrell’s C-index analysis of the comparison of the predictive accuracies of TNM staging and TMEM173 expression. (C) Nomogram for predicting clinical outcomes integrated TMEM173 expression (High/Low) with TNM classification (IA, IB, IIA, IIB, IV). In the nomogram, higher total point predicts worse prognosis. Addition of TNM classification (0 for “IA”, 16 for “IB”, 33 for “IIA”, 49 for “IIB” or 82 for “IV”) and TMEM173 expression (0 for “High” or 100 for “Low”) for each patient correspondingly gives the total point. (D) Calibration plot for nomogram predicted and observed 3-year survival rate. Calibration curves for nomogram predicted 3-year overall survival performed well with the ideal model. Line of dashes: ideal model; vertical bars, 95% confident interval. (E) Kaplan–Meier curves of overall survival based on risk score calculated by nomogram. P-value was assessed by log-rank test.

To establish a more sensitive prognostic model for HCC patients, we combined TMEM173 expression and TNM stages to create a prognostic score system. ROC curve analysis showed that the predictive value of TMEM173 alone (AUC [95% CI], 0.738 [0.616–0.860]) was higher than that of TNM stages (AUC [95% CI], 0.659 [0.537–0.782]). The combination of TMEM173 expression and TNM stages also revealed with statistical significance better prognostic value (AUC [95% CI], 0.800 [0.696–0.904]) than TNM stages alone (Fig 3A). In addition, the Harrell’s concordance index (C-index) for the combination of TNM stages and TMEM173 was 0.659, higher than that for TNM stages alone (0.589); the Akaike information criterion (AIC) was 554.64 when estimated according to TNM stages alone, and it decreased to 539.08 when estimated in combination with TMEM173 down-regulation (Fig 3B). These results suggest that combination of TMEM173 expression and TNM stages could establish a better prognostic model for the overall survival of HCC patients.

Based on the results of ROC analysis, we further constructed a nomogram model that integrated TNM classification with TMEM173 expression for better stratifying patients with differential prognoses. In this nomogram, a higher total point predicted a worse prognosis. The total point was raised by adding the score of the TNM classification (0 for “IA”, 16 for “IB”, 33 for “IIA”, 49 for “IIB” or 82 for “IV”) and TMEM173 expression (0 for “High” or 100 for “Low”) for each patient (Fig 3C). The calibration curve for predicting 3-year overall survival shows that the nomogram performs well with the ideal prediction model (Fig 3D). Based on the risk score, patients were stratified into three subgroups, including subgroups I for low risk score (<25%), subgroup II for medium risk score (25%-75%) and subgroup III for high risk score (>75%). Kaplan-Meier analysis revealed that scoring with the nomogram effectively discriminated the risk of postoperative survival in HCC patients (Fig 3E) (P < 0.001).

## Discussion

Various DNA viruses were reported to negatively regulate by the innate immune pathway via the activation of interferon response including HBV, Cytomegalovirus and Herpes Simplex Virus (HSV) as well as retroviruses such as Human Immunodeficiency Virus (HIV) [16]. And the detection pathway of exogenous DNA is essential in this process[16]. Recently, as one of the crucial adaptor proteins, TMEM173 was revealed to play a critical role in cytosolic DNA detection [8]. Meanwhile, accumulating studies demonstrated that the function of TMEM173 in immune surveillance was also involved in human cancers [10–12]. TMEM173 deficient mice bearing tumors showed shorter survival compared to wide-type controls[17]. And defective expression of TMEM173 was found in tumor tissues of colorectal carcinoma patients as well as peripheral blood mononuclear cells of HBV infected patients[11, 18]. In this study, we first identified the prognostic role of TMEM173 in HCC patients, and that loss of TMEM173 was positively associated with tumor progression and worse overall survival.

TMEM173 exerts its function by regulating dynamic cytokine expressions. Activation of TMEM173 signaling pathway results in cytokine production and subsequently immune cell recruitment. Type-I IFNs have been identified as the major effector in TMEM173-mediated anti-tumor immunity [9]. It is reported that type-I IFNs is essential for the maturation of CD11c+ CD8α+ DCs, and these DCs are critical for induction of tumor-reactive T-cell responses[19]. Type-I IFNs also contribute to the anti-tumor function of NK cells and macrophages [20]. Besides, TMEM173 can trigger IL-12 secretion that leads to proliferation and activation of CD8+ T cells and enhance CXCL10 production that recruits immune cells[21]. Therefore, loss of TMEM173 may not only contribute to HBV invasion of immune surveillance but also lead to defective anti-tumor immunity in HCC tumorigenesis. TMEM173 might also play a role in the anti-tumor effect through influencing checkpoint inhibitors such as CTLA4 and PD1[22]. Meanwhile, activation of TMEM173 could directly induce cell apoptosis and autophagy in malignant cells[23], which might inhibit tumor growth.

By the present study, it remains to be researched the underlying mechanism of TMEM173 expression in the progression of HCC. However, apart from tumor cells, extrinsic TMEM173 activity in tumor microenvironment was also likely to play a critical role in immune surveillance. Overall, the contribution of TMEM173 signaling in HCC remains far from being fully elucidated and need further investigation.

In conclusion, our study identified aberrant expression of TMEM173 in HCC as an independent prognostic factor, which could be integrated with TNM stages to generate a better risk stratification nomogram for HCC patients with differential prognoses. Future studies need to be focused on the underlying mechanisms of TMEM173 and related potential applications in the treatment of HCC.
